# Effects of domperidone in combination with omeprazole in the treatment of chronic superficial gastritis

**DOI:** 10.12669/pjms.332.11778

**Published:** 2017

**Authors:** Fengxiu Wang, Xiaoqi Zhang, Jinqiang Wang

**Affiliations:** 1 Fengxiu Wang, Pharmaceutical Department, Binzhou People’s Hospital, Shandong, 256600, China; 2 Xiaoqi Zhang, Pharmaceutical Department, Binzhou People’s Hospital, Shandong, 256600, China; 3 Jinqiang Wang, Pharmaceutical Department, Binzhou People’s Hospital, Shandong, 256600, China

**Keywords:** Chronic superficial gastritis, Domperidone, Omeprazole

## Abstract

**Objective:**

To find out the effects of domperidone in combination with omeprazole in the treatment of chronic superficial gastritis (CSG).

**Methods:**

Ninety-six patients who suffered from CSG and received treatment in the Binzhou People’s Hospital from July 2013 to July 2014 were selected as research subjects. They were divided into a control group (48 cases) and a test group (48 cases) using double blind method. Patients in the control group were treated by omeprazole, while patients in the observation group were treated by domperidone in combination with omeprazole. The clinical effects of the two groups were observed and analyzed.

**Results:**

The scores of symptoms had no significant difference between the two groups before treatment. The improvement of the scores of symptoms in the test group was superior to that in the control group after treatment (P<0.05). The overall response rate of the test group was 97.92% (47/48), higher than that of the control group (75.00%). After treatment, the repair effect of gastric mucosa and the postoperative recurrence rate in the test group were superior to those of the control group (P<0.05).

**Conclusion:**

Domperidone in combination with omeprazole can achieve ideal effect in the treatment of CSG, which is of great significance to the treatment and prognosis of patients.

## INTRODUCTION

Chronic superficial gastritis (CSG), a kind of chronic gastritis, is a commonly seen digestive system disease with a higher incidence. It is induced by chronic inflammatory lesions caused under the repeated actions of pathogenic factors such as microorganism, drugs and bile regurgitation on gastric mucosa epithelium. Its clinical symptoms include dyspepsia, stomachache, bloating, belching, nausea and emesis, which can produce severe impacts on the physical and psychological health and living quality.^1^,^2^ Epidemiological investigations demonstrated that, CSG had the highest incidence among all gastrointestinal diseases, which was 51.7%~85.45% that of chronic gastritis; the incidence increased with the increase of age; it might evolve to atrophic gastritis or gastric carcinoma if it was not treated timely.^3^-^5^ Thus, the early treatment of CSG and the inhibition of disease progress are meaningful to the prevention of gastric mucosa diseases.

A clinical trial^6^ demonstrated that, 50%~80% of patients who developed CSG had helicobacter pylori infection. Thus based on the onset mechanisms of chronic gastritis and relevant research data, the key treatment for CSG is to control helicobacter pylori infection and inhibiting excessive secretion of gastric acid. Currently, treating CSG with omeprazole has been a common therapy in clinics. Omeprazole as a proton pump inhibitor can produce selective effect on gastric mucosa wall cells, effectively inhibit tubular bubble inside cytoplasm and the activity of enzymes secreted by gastric wall cells, and finally inhibit the secretion of gastric acid; however, its effect is not quite satisfactory.^7^,^8^ A recent study^9^ indicated that, omeprazole in combination with domperidone was highly effective in treating CSG; but it has not been clarified clearly. To further clarify the effectiveness of the therapy, this study treated CSG patients with omeprazole in combination with domperidone, aiming to provide a scientific basis for clinical medication.

## METHODS

Ninety-six patients with CSG who received treatment in the Binzhou People’s Hospital from July 2013 to July 2015 and conformed to relevant diagnostic criteria were selected as research subjects. All of them were diagnosed by gastroscopic examination and pathological changes. They were divided into a control group and a test group using randomized double-blind method, 48 in each group. In the control group, there were 30 males and 18 females, with an average age of 42.8±2.7 years (14~82 years) and an average disease course of 1.2±0.2 years (2~13 months). In the test group, there were 27 females and 21 males, with an average age of 42.7±2.6 years (15~82 years old) and an average disease course of 1.4±0.4 years (2~11 months). The general data of patients in the two groups including gender, disease course and age had no remarkable difference (P>0.05); thus the results were comparable. All the patients were observed with dyspepsia and digestive system disorders. They had signed informed consent. They were forbidden to take other treatment drugs two weeks before this test. Patients with peptic ulcer or occupying lesions in digestive system were excluded.

Patients in the test group were treated by omeprazole (Actavis (Fushan) Pharmaceutical Co. Ltd., Guangdong, China; batch no.: H10930087) and domperidone (Xian Yangsen Pharmaceutical Co. Ltd., Shanxi; batch no.: H10910003). Domperidone was orally administrated in a dose of 10 mg each time 30 minutes before breakfast, lunch and supper; omeprazole was orally administrated in a dose of 20 mg each time in the morning and evening when patients were in the fasting state. Three weeks was regarded as one course.^10^ Patients in the control group orally took omeprazole, 20 mg each time in the morning and evening when patients were in the fasting state. The treatment lasted for three weeks. The patients were followed up and examined according to test procedures once each week after medication; the daily record of last week was recycled, and drugs that were needed in the next stage were issued to the patients.

### Observation index

Scores for the symptoms such as swelling pain, burning sensation and sour regurgitation of the upper abdomen were observed and recorded. The determination criteria for symptom scores were as follows. Mild symptoms were determined as 1~3 points, medium as 4~7 points, and severe as 8~10 points. The patients made self-assessment using visual analogue scale (VAS). The response rate of the two groups was compared. The determination criteria for curative effects were as follows. The complete disappearance of clinical symptoms and the disappearance of gastric mucosa inflammation observed by gastroscope were determined as cured. Treatment was determined as markedly effective if the clinical symptoms were obviously relieved and most of gastric mucosa inflammation disappeared. Treatment was determined as effective if some clinical symptoms still remained but relieved compared to before treatment and mucosal lesions reduced for more than 50% under gastroscope. Treatment was considered as ineffective if the symptoms showed no improvement or even aggravated and inflammation was not relived. The overall response rate was the sum of cure rate, markedly effective rate and effective rate. The last one was the repair effect of gastric mucosa, i.e., score of chronic inflammation and score of active inflammation. Lower score indicated better repair effect.

### Statistical analysis

Data were processed by SPSS ver. 20.0. Measurement data were expressed as mean ± SD and processed by t test. Enumeration data were expressed by percentage and processed by Chi-square test. Difference was considered as statistically significant if P<0.05.

## RESULTS

### Comparison of symptom scores

The scores of symptoms had no remarkable difference between the two groups before treatment (P>0.05). The scores for clinical symptoms such as swelling, pain, burning sensation and sour regurgitation of the upper abdomen showed a significant improvement in the test group compared to that of the control group (P<0.05) (Table-I).

**Table-I:** Comparison of symptom scores of the two groups before and after treatment (point, mean±SD)

*Group*	*Swelling pain*		*Burning sensation*		*Sour regurgitation*	

	*Before*	*After*	*Before*	*After*	*Before*	*After*
Test group	6.1±1.5	0.8±0.3*#	5.6±1.6	0.9±0.4*#	7.6±1.4	0.6±0.3*#
Control group	6.3±1.6	2.8±0.7#	5.4±1.8	3.2±1.3#	7.2±1.3	2.4±0.5#

**Note:**

* indicated P<0.05 compared to that of the control group;

# indicated P<0.05 compared to that before treatment.

### Comparison of clinical effects between the two groups

**Fig.1:**
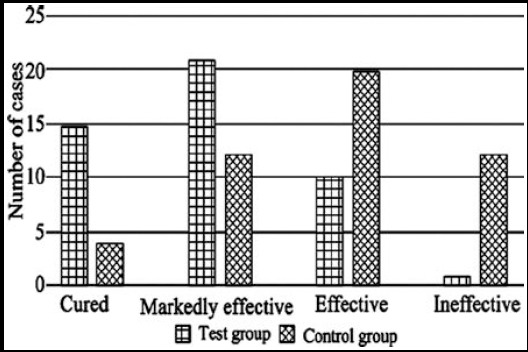
Comparison of clinical effects between the two groups.

The effective rate of the test group and the control group was 97.92% and 75.00% respectively, and there was a remarkable difference between the two groups (P< 0.05) (Fig.1).

### Comparison of gastric mucosa repair between the two groups

After treatment, the scores of chronic inflammation and active inflammation of the test group were higher than those of the control group, and there was an obvious difference (P<0.05) (Table-II), indicating gastric mucosa of the test group repaired better than that of the control group. It showed that, the therapy of the test group was more effective in relieving the disease condition.

**Table-II:** Comparison of gastric mucosa repair between the two groups after treatment (point, mean±SD).

*Group*	*N*	*Score of chronic inflammation*	*Score of active inflammation*
Test group	48	1.02±0.13	1.03±0.22
Control group	48	1.63±0.44	1.68±0.37
t		4.872	5.273
P		<0.05	<0.05

### Comparison of adverse reactions and recurrence between the two groups

During treatment, the symptoms of lacking in strength and dizziness occurred in two cases in the control group (4.17%); in the test group, no case had adverse reactions (0%). The incidence of adverse reactions of the two groups showed no significant difference (P>0.05). The patients in the two groups were followed up for six months. One case in the test group and nine cases in the control group recurred (2.08% vs 18.75%). The recurrence rate of the test group was lower than that of the control group, and there was a statistically significant difference (P<0.05).

## DISCUSSION

The symptoms of patients with CSG would disappear after medication, but usually recur repeatedly. CSG is induced by chronic inflammatory lesions on gastric mucosa epithelium under the stimulation of pathogenic factors. The main pathogenic factors of CSG include toxin, microorganism, bile regurgitation and drugs. The causes for pathogenic factors are complex; hence it is difficult to make accurate diagnosis during treatment, which leads to difficulty in the early prevention of CSG.^11^ With the constant development of clinical medicine, drugs for treating CSG have increased, but clinical effects have large difference. Therefore, the key of its clinical treatment is to select safe and effective treatment drugs and methods.

In the occurrence of CSG, the erosion effect of gastric acid plays an important role. Omeprazole as a kind of white crystalline powder can reduce the activity of H^+^, K^+^ and APT enzyme, inhibit the secretion of gastric acid and prevent bacterial infection by acting on gastric mucosal wall cells directly. Omeprazole can be distributed in the secretory tubules in gastric mucosal walls through blood circulation and block the secretion of gastric acid by inhibiting the activity of proton pump through combining with it.^12^ In the process of gastric acid secretion inhibition, it can reduce the evacuation of drugs, improve drug utilization rate, and effectively relieve clinical symptoms of patients; hence it has been extensively applied in clinical treatment of chronic gastritis. In addition, omeprazole can combine with urease to inhibit the activity of urease and eliminate Hp by penetrating mucous layer and Hp surface.^13^ Therefore, it is frequently used to treat CG. However, a study^14^ demonstrated that, omeprazole could be used for treating CSG, but its effect was not satisfactory. The results of this study demonstrated that, 12 cases out of the 48 cases in the control group showed no response to the treatment using omeprazole, with an efficacy rate of 75.00%, which was consistent with relevant research results.

Domperidone as a kind of peripheral dopamine receptor inhibitor can resist emesis. It will not produce bad effects on the central nervous system as it is difficult to enter the brain. Besides, it can selectively block dopamine 2 and mainly act on the peripheral nervous system.^15^ Domperidone can improve the power of digestive tract and promote gastric emptying.^16^ Compared to metoclopramide and cisapride, domperidone was safer and extensively applied. In the treatment of chronic gastritis cannot inhibit gastric acid and protect gastric mucosa. But omeprazole in combination with domperidone can give full play to the acid and helicobacter pylori inhibition effects of omeprazole as well as the emesis resistance and gastric emptying effect of domperidone. The two drugs can act on the different clinical symptoms of CSG, with remarkable effect and less reverse reactions. The results of this study demonstrated that, the overall effective rate of the test group was higher than that of the control group, and the difference had statistical significance (P<0.05). The scores of clinical symptoms such as swelling pain, burning sensation and sour regurgitation of the test group had better improvement compared to the control group; the gastric mucosa repair effect and the postoperative recurrence rate of the test group were both superior to those of the control group, and the difference was statistically significant (P<0.05). All the findings suggested, the combined therapy could significantly improve the clinical symptoms, repair gastric mucosa, and improve treatment effect, with a high safety.

## CONCLUSION

In conclusion, omeprazole in combination with domperidone was highly effective in treating CSG, with simple operation and high safety. It can accelerate cure and obviously relieve the clinical symptoms of patients. Hence it can be regarded as a preferred method for treating CSG in clinics.

### Authors’ Contribution

**FXW:** Study design, data collection and analysis.

**XQZ and JQW:** Manuscript preparation, drafting and revising.

**FXW:** Review and final approval of manuscript.
